# Trajectories of Social Isolation and Loneliness and Advance Care Planning among U.S. Older Adults

**DOI:** 10.1093/geroni/igaf122.3677

**Published:** 2025-12-31

**Authors:** Ke Li, Yifan Lou, Jing Wang

**Affiliations:** Ohio University, Athens, Ohio, United States; Virginia Commonwealth University, Richmond, Virginia, United States; University of New Hampshire, Durham, New Hampshire, United States

## Abstract

Social isolation and loneliness are critical public health concerns that may interfere with end-of-life care preferences. However, their relationships with advance care planning (ACP) remain unclear. Using three-wave data from the Health and Retirement Study, this study identified trajectory patterns of social isolation and loneliness and explored their associations with ACP. Social isolation index was generated using five indicators of marital status, social participation, and frequency of contact. Loneliness was measured by the UCLA loneliness scale. Three dimensions of ACP were considered: living will, Durable Power Attorney for Health Care (DPAHC), and ACP discussions. Group-based trajectory model identified four distinct trajectories for both social isolation (low start-decreasing, low start-increasing, moderate-start stable, high start-stable) and loneliness (low start-stable, low start-increasing, moderate start-decreasing, high start-stable). The results of logistic regression models indicated that both trajectories were significantly related to discussion and DPAHC, but not living will. The high start-stable isolation group had the lowest odds of having discussion across groups and lower odds of having DPAHC than those with moderate start-stable isolation. Compared to low start-stable loneliness group, low start-increasing loneliness group was less likely to have DPAHC. The odds of having discussion were significantly lower among the groups of high start-stable loneliness and low start-increasing loneliness than those with low start-stable loneliness. Gender-specific analyses showed that females were affected by both social isolation and loneliness, whereas males were solely affected by social isolation. The findings highlight the importance of developing targeted interventions to address social isolation and loneliness to increase ACP.

